# Experiencing, anticipating, and witnessing discrimination during the COVID-19 pandemic: Implications for health and wellbeing among Asian Americans

**DOI:** 10.3389/fpubh.2022.949403

**Published:** 2022-10-13

**Authors:** Lindsay Y. Dhanani, Berkeley Franz, Carolyn T. Pham

**Affiliations:** ^1^School of Management and Labor Relations, Rutgers University, Piscataway, NJ, United States; ^2^Heritage College of Osteopathic Medicine, Appalachian Institute to Advance Health Equity Science, Ohio University, Athens, OH, United States; ^3^Psychology Department, DePaul University, Chicago, IL, United States

**Keywords:** Asian Americans, COVID-19, discrimination, vigilance, health

## Abstract

The onset of the COVID-19 pandemic spurred increased racial animus toward Asians and Asian Americans (A/AA) who have since been contending with increased racism and violence. While some of the harm associated with this increased prejudice may derive from personally experienced discrimination, the COVID-19 pandemic has also been marked by an increase in vicarious exposure to discrimination as well as increased anticipation of discrimination, both of which may be taxing for the mental and physical health of A/AA. The goal of this study, accordingly, was to examine the effects of personal experiences of discrimination, vicarious exposure to discrimination, and anticipated discrimination on depressive symptoms, physical health symptoms, sleep quality, and sleep disturbances among A/AA. Results from our two-wave field survey demonstrated that experiencing and anticipating discrimination were associated with mental and physical health symptoms as well as sleep disturbances. Further, personal experiences of discrimination interacted with vicarious discrimination to determine physical health symptoms such that greater vicarious exposure weakened the relationship between experienced discrimination and physical health symptoms. These findings demonstrate the need to mobilize resources to combat the multipronged, negative implications of the recent rise in anti-Asian prejudice during the COVID-19 pandemic.

## Introduction

Since the onset of the COVID-19 pandemic, reports of discrimination and violence against Asians and Asian Americans (A/AA) have increased substantially across the United States (1, 2). Although overall hate crimes decreased by 7% nationwide, hate crimes targeting A/AA rose by 150% in 2020 and further increased in 2021 (2, 3). More than 10,000 hate crimes were self-reported during this same time period (2). Further, roughly 39% of A/AA reported experiencing discrimination at least sometimes or often during the pandemic and more than half of A/AA reported feeling unsafe in public due to their race/ethnicity (4).

Although discrimination toward A/AA has a long history in the United States, the inflammatory rhetoric linking the origin of the novel coronavirus to China contributed to a further rise in discrimination among A/AA which has important implications for health outcomes. Specifically, studies have indicated that derogatory names used in place of COVID-19 in mainstream media messaging as well as on social media are associated with prejudice toward A/AA (1, 5–7). Researchers have also observed “spillover” effects in which discrimination is targeted at other A/AA groups beyond Chinese Americans (5, 8). Yet, limitations in data reporting on A/AA have complicated our ability to understand the full scope of health and social threats faced by A/AA and it is likely that current estimates of hate crimes are an undercount (9).

Although data are limited on the health implications resulting from the rise in A/AA discrimination, extant empirical evidence confirms the linkage between experiencing discrimination among A/AA during the COVID-19 pandemic and a range of mental health outcomes, including PTSD diagnoses (10), depressive symptoms (11), and sleep quality (12). These findings comport with more general evidence documenting the psychological and physiological effects of experiencing discrimination. Indeed, the well-known weathering hypothesis (13) suggests that long term exposure to discrimination and various forms of social disadvantage contributes to accelerated biological aging and a higher chronic disease burden [e.g., (14)]. Importantly, even more subtle or chronic forms of interpersonal discrimination, such as microaggressions and displays of favoritism, may trigger this physiological stress response which over time can degrade health status, increasing rates of hypertension and both maternal and infant mortality (15, 16), among other conditions. These figures suggest that direct experiences of discrimination incurred by A/AA during the COVID-19 pandemic likely contribute to significant short and long-term health consequences, above and beyond the threats posed by the pandemic itself.

There may be other ways, however, that periods of increased racial animus, such as what has been observed in response to the COVID-19 pandemic, can shape the health of A/AA. That is, the acute rise in anti-Asian discrimination not only increased the likelihood that A/AA will experience discrimination directly but may have also heightened vicarious exposure to discrimination as well as anticipation of discrimination. Vicarious exposure to discrimination can occur through witnessing discrimination targeted at other A/AA while in public, hearing experiences detailed by friends or family members, or reading about violence and discrimination targeting A/AA in the news. Being aware of these events and the general rise in anti-Asian sentiment can then also spur increased vigilance, or a heightened anticipation that one may also become a target of anti-Asian discrimination. In this paper, we argue that vicarious discrimination and the anticipation of discrimination may each uniquely contribute to ill health among A/AA, above and beyond personal experiences of discrimination.

Prior empirical evidence supports the linkage between vicarious exposure to discrimination and adverse health outcomes. Studies suggest that witnessing discrimination directly, or living in communities with high levels of discrimination, also contributes to poor health through activating the stress process. For example, living in areas with higher rates of hate crimes is associated with poor physical health outcomes, including hypertension, diabetes, and obesity (17). Similarly, witnessing discrimination during childhood has been linked to a broad array of socioemotional and mental health consequences (18). Extant research on vigilance demonstrates that anticipating discrimination is associated with sleep impairment (19), obesity (20), and depressive symptoms (21).

The goal of the current study is to examine how experienced discrimination, vicarious discrimination, and vigilance during the COVID-19 pandemic relate to mental health and physical health symptoms, and indices of sleep using a two-wave field survey of A/AA. The current study makes three primary contributions. First, we contribute to the small but growing body of literature on the health decrements associated with vicarious discrimination and vigilance. These novel forms of discrimination are important given the profound health inequities observed across racial/ethnic groups in the U.S. Second, studies on the health consequences of interpersonal discrimination have commonly centered around Black and Latinx Americans, long overlooking the experiences of other racial/ethnic minorities in the United States, including A/AA. Scholars point to the “model minority myth” which suggests that A/AA are universally successful and free of problems, contributing to a tendency to discount discrimination and its consequences among this group (22, 23). Third, this study examines health outcomes associated with different forms of discrimination during the COVID-19 pandemic, a period in which marked increases in interpersonal violence toward A/AA were documented (24, 25).

## Method

Data were collected from people who identified their race/ethnicity as Asian American, were 18 years of age or older, and resided within the United States at the time of data collection. Participants were recruited through Qualtrics Panels, which is a third-party research firm that connects eligible participants to research surveys. Qualtrics Panels utilizes existing survey platforms to recruit participants who meet specified criteria. As such, our participants were limited to registered members of existing online research platforms. In addition to the eligibility criteria listed above, we also recruited a sample that was geographically dispersed across the United States and balanced in terms of sex at Time 1. The data collection occurred in April 2021 and participants were asked to complete two surveys that were separated by ~1 week of time. A time lag was implemented to reduce concerns about common method variance, which can upwardly bias correlations between/among measures collected at the same time (26). The Time 1 survey contained measures of experienced discrimination, vicarious exposure to discrimination, vigilance, and participant demographics. The Time 2 survey contained the outcome measures, which included mental health, physical health, sleep quality, and sleep disturbances.

A total of 401 eligible participants provided data at Time 1 and 311 of those participants also provided data at Time 2. Among participants who completed both surveys, the average age was 33.38 (*SD* = 8.50) and approximately half identified as women (50.2%). The majority of respondents identified their gender as cisgender, with only two participants identifying as transgender or genderqueer. The most commonly reported sexual orientation was heterosexual (92.3%) followed by bisexual (3.5%), other (2.3%), and gay/lesbian (1.9%). To assess for nonresponse bias, we conducted a series of *t*-tests that compared participants who completed both surveys to those who only completed the Time 1 survey on age, sex, work hours, experienced discrimination, vicarious discrimination, and vigilance. Results indicated no significant differences between groups.

### Measures

#### Experienced discrimination (T1)

We measured experiences of racial discrimination since the onset of the COVID-19 pandemic using the Everyday Discrimination Scale [(27); α = 0.96]. This measure contains 9 items that ask about experiences of discrimination that can occur in a variety of commonplace interactions and contexts, such as when someone is in public or in restaurants. The measure was developed for use with a variety of social groups and has been used with Asian Americans in prior research (28, 29). An example item is, “You were treated with less courtesy than other people were.” The response scale ranged from 1 (*never*) to 6 (*almost every day*) and a scale score was created by taking an average across all items.

#### Vigilance (T1)

Participants were also asked to report their vigilance, or the degree to which they have anticipated and/or tried to prepare for experiences of racial discrimination, since the onset of the COVID-19 pandemic. We used Hicken et al. (20)'s 4-item measure of vigilance and adapted the scale to be specific to experiences of anti-Asian racial discrimination. The modifications aligned with another recent study which examined vigilance among Asian Americans (30). An example item is, “Try to prepare for possible racial insults from other people before leaving home.” Participants rated the frequency with which they experienced vigilance on a scale ranging from 1 (*never*) to 6 (*almost every day*) and a scale score was created by taking an average across all items. The scale demonstrated good reliability in the current sample (α = 0.88).

#### Vicarious discrimination (T1)

Participants were finally asked to report on their experiences of vicarious discrimination, or instances in which they witnessed or learned about other A/AA experiencing racial discrimination, since the onset of the COVID-19 pandemic. Vicarious discrimination was measured using an adapted version of the 3-item Vicarious Racism Scale developed by Martz et al. (31). The original items were modified to be specific to acts of anti-Asian racism and/or violence. A recent study found this measure was appropriate for use with Asian Americans and Pacific Islanders (32). An example item from the adapted scale is, “Seeing other people in public being treated unfairly because they are Asian.” Participants indicated the frequency with which they had been exposed to each form of vicarious racism on a scale ranging from 1 (*never*) to 6 (*almost every day*) and a scale score was created by taking an average across all items. The modified scale demonstrated adequate reliability (α =0.71).

#### Depressive symptoms (T2)

At Time 2, participants were asked to report on several indicators of their health and wellbeing. The first indicator was the degree to which they had experienced symptoms commonly associated with depression. Depressive symptoms were measured using the shortened Patient Health Questionnaire [PHQ-9; (33)]. This measure is often used in clinical contexts as a brief but effective way to screen for depression. The measure typically contains 9 items, but we used an 8-item version of the scale that removed the single item that asked about suicidality. The retained items ask about typical symptoms of depression, such as experiencing a loss of interest in activities and feeling hopeless. Participants were asked to indicate how bothered they were by those symptoms since completing the Time 1 survey on a scale from 0 (*not at all*) to 3 (*nearly every day*) and a scale score was created by taking an average across all items. The scale demonstrated good reliability (α = 0.90).

#### Physical health (T2)

Participants were next asked about their physical health using the Physical Health Questionnaire [(34); α = 0.89]. The Physical Health Questionnaire contains a series of physical health symptoms that are commonly associated with somatic reactions to stressful experiences, such as headaches and gastrointestinal symptoms. The scale consisted of 11 items containing physical health symptoms and response options ranged from 1 (*not at all*) to 7 (*all of the time*). A scale score was created by taking an average across all items.

#### Sleep (T2)

We next assessed participants' sleep in two different ways. First, we asked participants to rate their sleep quality using a single item (i.e., During the past week, how would you rate your sleep quality overall?). Second, we asked participants about their experiences of sleep disturbances. Participants were asked to indicate the frequency with which they experienced ten common types of sleep disturbances, including not being able to fall asleep and not being able to stay asleep. Response options ranged from 1 (*never*) to 4 (*three or four times per week*) and a scale score was created by summing the total number of sleep disturbances participants experienced. Both the sleep quality and sleep disturbance items were taken from the Pittsburgh Sleep Quality Index (35).

### Analyses

Study hypotheses were tested using ordinary least squares multivariate regression. Each model included participant demographics (i.e., age, sex [coded 1 = male, 2 = female], sexual orientation [coded 1 = heterosexual, 2 = gay, lesbian, bisexual, or pansexual) in Step 1 and the focal predictor variables (i.e., experienced discrimination, vicarious discrimination, vigilance) in Step 2, and separate models were conducted for each of the outcome variables. Demographics were included as control variables given prior evidence which suggests that health and wellbeing outcomes differ across demographic subgroups (36). Supplemental analyses were conducted to examine whether there were interactions between experienced discrimination and vicarious discrimination as well as between experienced discrimination and vigilance. Interactions were tested by computing product terms from the relevant variables and entering the product term in Step 3 of the regression model. To reduce concerns about multicollinearity, all continuous predictor variables were mean-centered and product terms were computed from the centered variables. Significant interactions were interpreted by calculating the simple slopes for the relationship between one predictor variable and the outcome at one standard deviation above and below the mean of the second predictor variable (37).

## Results

Descriptive statistics for the study variables are shown in Table 1. An examination of the correlations indicated that experienced discrimination was significantly positively related to depressive symptoms (*r* = 0.31, *p* < 0.001), physical health symptoms (*r* = 0.32, *p* < 0.001), and sleep disturbances (*r* = 0.23, *p* < 0.001). Vigilance was similarly related to depressive symptoms (*r* = 0.35, *p* < 0.001), physical health symptoms (*r* = 0.38, *p* < 0.001), sleep quality (*r* = −0.21, *p* < 0.001), and sleep disturbances (*r* = 0.20, *p* < 0.001). Finally, vicarious discrimination was also significantly related to all of the outcome variables (depressive symptoms: *r* = 0.25, *p* < 0.001; physical health symptoms: *r* = 0.27, *p* < 0.001; sleep quality: *r* = −0.17, *p* = 0.003; sleep disturbances: *r* = 0.20, *p* < 0.001). These results indicate that experiencing discrimination, anticipating discrimination, and being vicariously exposed to discrimination was associated with increased depressive symptoms, increased physical health symptoms, and increased sleep disturbances. Further, vicarious discrimination and vigilance were also associated with reduced sleep quality.

**Table 1:** Reliabilities appear on the diagonal.

	**M**	**SD**	**1**	**2**	**3**	**4**	**5**	**6**	**7**	**8**	**9**	**10**
1. Age	33.57	8.51										
2. Sex	1.50	0.50	−0.22^*^									
3. Sexual orientation	1.08	0.27	−0.13^*^	0.05								
4. Experienced discrimination	1.94	1.17	−0.10	−0.02	0.05	*0.96*						
5. Vigilance	3.28	1.57	−0.08	0.09	−0.03	0.29^*^	*0.88*					
6. Vicarious discrimination	3.06	1.21	−0.11	0.04	−0.02	0.38^*^	0.61^*^	*0.71*				
7. Depressive symptoms	1.91	0.65	−0.18^*^	0.12^*^	0.08	0.31^*^	0.35^*^	0.25^*^	*0.90*			
8. Physical health symptoms	3.07	1.09	−0.12^*^	0.15^*^	0.03	0.32^*^	0.38^*^	0.27^*^	0.66^*^	*0.89*		
9. Sleep quality	2.57	0.71	−0.01	0.00	−0.06	−0.10	−0.21^*^	−0.17^*^	−0.47^*^	−0.55^*^	—	
10. Sleep disturbances	22.14	6.69	0.03	0.06	−0.02	0.31^*^	0.23^*^	0.20^*^	0.60^*^	0.73^*^	−0.53^*^	—

*N* = 311; Sex is coded 1 = male, 2 = female; sexual orientation is coded 1 = heterosexual, 2 = gay, lesbian, bisexual, or pansexual; reliability coefficients are presented on the diagonal.

* p < 0.05.

### Regression analyses

Results for the regression models are shown in Table 2. Results for the first model, which included depressive symptoms as the outcome variable, demonstrated a significant negative relationship between depressive symptoms and age (*b* = −0.01, 95% CI [−0.20, −0.002], *p* = 0.015). There were also significant positive relationships between experienced discrimination and depressive symptoms (*b* = 0.13, 95% CI [0.07, 0.19], *p* < 0.001) as well as between vigilance and depressive symptoms (*b* = 0.12, 95% CI [0.07, 0.17], *p* < 0.001). However, the relationship between vicarious discrimination and depressive symptoms was not significant. This suggests that experienced discrimination and vigilance were both associated with increased depressive symptoms. The second model predicted physical health symptoms as the outcome variable. In Step 1, sex had a significant positive relationship with physical health symptoms (*b* = 0.28, 95% CI [0.03, 0.53], *p* = 0.027), indicating women reported more physical health symptoms than men. There were additionally significant positive relationships for experienced discrimination (*b* = 0.22, 95% CI [0.12, 0.32], *p* < 0.001) and vigilance (*b* = 0.20, 95% CI [0.12, 0.29], *p* < 0.001). However, there was again no significant relationship between vicarious discrimination and physical health symptoms. Results demonstrate that experiencing discrimination and vigilance were associated with heightened physical health symptoms.

**Table 2:** Experienced discrimination, vigilance, and vicarious discrimination predicting health and wellbeing outcomes.

	**Depressive symptoms**			**Physical health**			**Sleep quality**			**Sleep disturbances**		
**Variable**	**B**	**SE**	**(Δ)R^2^**	**B**	**SE**	**(Δ)R^2^**	**B**	**SE**	**(Δ)R^2^**	**B**	**SE**	**(Δ)R^2^**
**Step 1**												
Sex	−0.01^*^	0.01		−0.01	0.01		0.00	0.01		0.03	0.05	
Age	0.10	0.08		0.28^*^	0.13		0.00	0.08		0.80	0.78	
Sexual orientation	0.13	0.14		0.03	0.23		−0.17	0.15		−0.59	1.44	
			0.038			0.029			0.004			0.005
**Step 2**												
Experienced discrimination	0.13^***^	0.03		0.22^***^	0.05		−0.02	0.04		1.54^***^	0.33	
Vigilance	0.12^***^	0.03		0.20^***^	0.05		−0.08^*^	0.03		0.59^*^	0.29	
Vicarious discrimination	−0.01	0.04		0.00	0.06		0.04	0.04		0.11	0.39	
			0.160			0.177			0.049			0.120
**Step 3**												
Experienced discrimination × Vicarious discrimination	−0.03	0.03		−0.13^**^	0.05		0.01	0.04		−0.50	0.31	
Experienced discrimination × Vigilance	−0.02	0.03		−0.03	0.04		0.01	0.03		−0.23	0.26	
			0.010			0.030			0.001			0.017

N = 301 for the models predicting depressive symptoms, 308 for the models predicting physical health, sleep quality, and sleep disturbances; Sex is coded such that 1 = male and 2 = female; Sexual orientation is coded such that 1 = heterosexual and 2 = gay, lesbian, bisexual, or pansexual; All continuous predictor variables are mean centered.

* p < 0.05,

** p < 0.01,

*** p < 0.001.

The final two models predicted sleep quality and sleep disturbances. Beginning with sleep quality, results showed that the only significant predictor of sleep quality was vigilance (*b* = −0.08, 95% CI [−0.14, −0.01], *p* = 0.018), with more vigilance relating to lower sleep quality. For sleep disturbances, there were significant relationships for experienced discrimination (*b* = 1.54, 95% CI [0.88, 2.20], *p* < 0.001) and vigilance (*b* = 0.59, 95% CI [0.02, 1.16]), *p* = 0.042). This suggests that experiencing and anticipating more discrimination were associated with increased sleep disturbances. In contrast, vicarious discrimination was unrelated to sleep disturbances.

### Supplemental analyses

**Figure 1:**
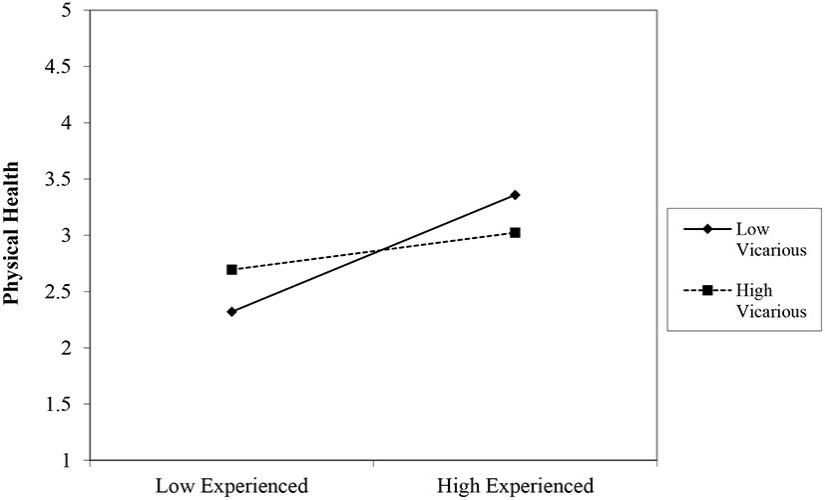
Interaction of experienced discrimination and vicarious discrimination on physical health. *N* = 308. Experienced, experienced discrimination; Vicarious, vicarious discrimination.

We additionally considered interactions between experiencing discrimination and either witnessing discrimination directed toward others or anticipating discrimination. Previous work has contended that the harms of experiencing discrimination may be compounded by other, similar identity-related stressors such as seeing discrimination directed toward others who hold a shared social identity (38–40). To assess that possibility, we computed interaction terms for experienced discrimination and vigilance and for experienced discrimination and vicarious discrimination. The relevant interaction terms were entered in Step 3 of the previous regression models. Results indicated only one significant interaction between experiencing discrimination and vicarious exposure to discrimination for physical health symptoms (*b* = −0.13, 95% CI [−0.22, −0.03], *p* = 0.010). We probed this interaction by plotting the relationship between experienced discrimination and physical health symptoms and one standard deviation above and below the mean for vicarious discrimination (see Figure 1). Results showed that the relationship between experienced discrimination and physical health is stronger when vicarious discrimination is low (*b* = 0.44, *p* < 0.001) as compared to when vicarious discrimination is high (*b* = 0.14, *p* = 0.019).

## Discussion

The aim of the current study was to examine the impact of experienced and vicarious discrimination as well as vigilance on depressive symptoms, physical health symptoms, and sleep outcomes among A/AA during the COVID-19 pandemic. Results suggested that both experiencing and anticipating discrimination were associated with poor physical health, mental health, and sleep-related outcomes independent of demographic factors. This is in line with previous research utilizing the racism-stress framework, which identifies psychosocial stressors of racism, such as experienced discrimination and vigilance, as key mechanisms by which racial/ethnic health disparities are reproduced (19, 41, 42). However, the consequences of vigilance, or the anticipation of negative treatment, have seldom been examined in the extant literature, particularly among A/AA.

Although we did not find a direct relationship between vicarious discrimination and health outcomes, we found a significant interaction between experiencing and witnessing discrimination for physical health symptoms. These results suggested that vicarious discrimination can have a buffering effect on physical health outcomes when one also experiences discrimination. Though seemingly counterintuitive, this finding is consistent with previous work suggesting that the health effects of experiencing discrimination may be lessened when individuals also witness discrimination against others [e.g., (43)]. Observing discrimination may signal that one is not being uniquely targeted by discrimination and may facilitate external attributions. Indeed, empirical evidence has demonstrated that people experience lower self-blame after experiencing mistreatment when they have also witnessed mistreatment toward others (43). Our finding that vicarious discrimination was unrelated to health outcomes, however, deviates from results from other recent studies (30, 44). Importantly, though, our study differed from the other studies in that it controlled for other types of discrimination experiences (e.g., personally experienced discrimination). It is therefore possible that the effects of vicarious discrimination were eliminated after controlling for other discrimination exposure.

### Public health implications

Our findings underscore the importance of assessing multiple types of exposure to discrimination when examining the public health outcomes associated with the COVID-19 pandemic. Even after accounting for personally experienced and vicarious discrimination, our study highlights that vigilance can also erode the health and wellbeing of A/AA. Vigilance remains an understudied type of discrimination, particularly among A/AA. Our findings underscore the need for future empirical studies to incorporate vigilance into efforts to improve wellbeing among minoritized communities.

Although vicarious discrimination was not directly related to the health outcomes examined in this study, vigilance was strongly positively related to vicarious exposure to discrimination. This finding suggests that witnessing discrimination directed toward A/AA may signal that one needs to prepare themselves to also experience discrimination. Moreover, we found that vicarious discrimination interacted with experienced discrimination to determine physical health symptoms such that witnessing discrimination toward others buffered the impact of personally experiencing discrimination. We issue caution in interpreting this as a potentially positive consequence of witnessing discrimination; indeed, the correlational analyses confirm that vicarious discrimination is harmful for a host of health outcomes. However, practitioners can use this finding to recognize the importance of facilitating external attributions when attempting to reduce the harm of discrimination. This aligns with previous findings that uphold the mitigating effects of external attributions following adverse treatment (45).

In sum, health experts should be aware of multiple and unique forms of discrimination and their potential to compromise the health and wellbeing of A/AA. Focusing only on experienced discrimination may fail to capture important social determinants of A/AA health.

### Limitations and future research

Although this study incorporated a time lag between the predictor and outcome variables to reduce concerns about common method variance, we were unable to establish causality. However, the direction of the relationships examined in this study comport with prior longitudinal studies on discrimination and health [e.g., (46)], which support that experienced and anticipated discrimination are antecedent to poor health outcomes. Moreover, our measures of experienced, anticipated, and vicarious discrimination focused on general discriminatory behaviors directed toward A/AA that occurred during the COVID-19 pandemic rather than discriminatory behaviors that are specific to the COVID-19 pandemic (e.g., blaming A/AA for the spread or origin of COVID-19). We adopted this approach because COVID-19 is more likely to increase the frequency of discrimination directed toward A/AA rather than alter the specific expression of prejudice. However, it is possible that A/AA also experienced unique forms of discrimination during the COVID-19 pandemic that are also worthy of study.

Next, sleep quality was assessed using a single-item measure which may have contributed to the relatively weak observed relationships with the three forms of discrimination. Though measuring sleep quality in this way is common, it is possible that our measure deflated relationships. Subsequent research should continue to explore the relationship between discrimination and perceived sleep quality to clarify these findings. We also did not consider differences based on the specific ethnicity of our participants and it is possible that exposure to discrimination operated differently across subgroups of A/AA. For example, Chinese Americans may experience higher levels of discrimination and/or more severe consequences in the context of the COVID-19 pandemic. We recommend that future research consider this possibility as well as specific ways to reduce vigilance. Current work has conceptualized the conditions that might result in vigilance, but little attention has been devoted to identifying ways to lessen its impact on health.

## Conclusion

The current study extends previous work on the rise in anti-Asian discrimination observed during the COVID-19 pandemic and its impact on health by considering two novel forms of discrimination: vicarious exposure to discrimination and vigilance or anticipated discrimination. Results demonstrated that experiencing discrimination and anticipating discrimination were each uniquely associated with negative mental and physical health symptoms as well as disrupted sleep. We found an interactive effect found between experienced discrimination and vicarious discrimination on physical health, indicating that both experiences combine to affect physical health symptoms. Public health officials and scholars can leverage these findings to better understand the full range of social determinants that may be harming the wellbeing of A/AA, including as part of the COVID-19 pandemic, and develop corresponding interventions to protect A/AA health.

## Data availability statement

The raw data supporting the conclusions of this article will be made available by the authors, without undue reservation.

## Ethics statement

The studies involving human participants were reviewed and approved by Ohio University Institutional Review Board. The patients/participants provided their written informed consent to participate in this study.

## Author contributions

LD contributed to study planning, data collection, analyses, writing, and editing. BF contributed to analyses, writing, and editing. CP contributed to study planning, writing, and editing. All authors contributed to the article and approved the submitted version.

## Conflict of interest

The authors declare that the research was conducted in the absence of any commercial or financial relationships that could be construed as a potential conflict of interest.

## Publisher's note

All claims expressed in this article are solely those of the authors and do not necessarily represent those of their affiliated organizations, or those of the publisher, the editors and the reviewers. Any product that may be evaluated in this article, or claim that may be made by its manufacturer, is not guaranteed or endorsed by the publisher.
